# Cyclooxygenase-2 in tumor-associated macrophages promotes breast cancer cell survival by triggering a positive-feedback loop between macrophages and cancer cells

**DOI:** 10.18632/oncotarget.4936

**Published:** 2015-08-10

**Authors:** Hongzhong Li, Bing Yang, Jing Huang, Yong Lin, Tingxiu Xiang, Jingyuan Wan, Hongyuan Li, Salem Chouaib, Guosheng Ren

**Affiliations:** ^1^ Chongqing Key Laboratory of Molecular Oncology and Epigenetics, The First Affiliated Hospital of Chongqing Medical University, Chongqing, China; ^2^ Department of Pharmacology, Chongqing Medical University, Chongqing, China; ^3^ Unite INSERM U753, Institut de Cancerologie Gustave Roussy, Paris, France

**Keywords:** tumor microenvironment, macrophages, breast cancer, cyclooxygenase-2, prostaglandin E_2_

## Abstract

Tumor-associated macrophages (TAMs) play an important role in cancer cell survival, however, the mechanism of which remains elusive. In this study, we found that COX-2 was abundantly expressed in breast TAMs, which was correlated to poor prognosis in breast cancer patients. Ectopic over-expression of COX-2 in TAMs enhanced breast cancer cell survival both *in vitro* and *in vivo*. COX-2 in TAMs was determined to be essential for the induction and maintenance of M2-phenotype macrophage polarity. COX-2^+^ TAMs promoted breast cancer cell proliferation and survival by increasing Bcl-2 and P-gp and decreasing Bax in cancer cells. Furthermore, COX-2 in TAMs induced the expression of COX-2 in breast cancer cells, which in turn promoted M2 macrophage polarization. Inhibiting PI3K/Akt pathway in cancer cells suppressed COX-2^+^ TAMs-induced cancer cell survival. These findings suggest that COX-2, functions as a key cancer promoting factor by triggering a positive-feedback loop between macrophages and cancer cells, which could be exploited for breast cancer prevention and therapy.

## INTRODUCTION

The tumor microenvironment comprises a variety of stromal cells that play an essential role in tumor initiation and progression [1]. Tumor associated macrophages (TAMs), the most abundant inflammatory stromal cells in malignant tumors including breast cancer, have been implicated in orchestration of many stages of tumor progression such as tumor growth, angiogenesis, metastasis, and resistance to treatment, through releasing various factors including chemokines, inflammatory and growth factors [2, 3]. Several studies have indicated that increased infiltration of TAMs to breast cancer is strongly associated with poor prognosis in patients [4, 5].

Macrophages have a tremendous plasticity and can change their functional profiles repeatedly in response to environmental stimuli. When exposed to lipopolysaccharides (LPS) and IFN-γ, macrophages are polarized to proinflammatory M1 (classical) macrophages and exert strong microbicidal and tumoricidal activities. Conversely, when exposed to Th2 cytokines such as IL-4 and IL-13, they are polarized to immunosuppressive M2 macrophages and involved in parasite containment, tissue remodeling and tumor progression. Compared with M1 macrophages, M2 macrophages do not produce pro-inflammatory mediators such as tumor necrosis factor-α (TNF-α), IL-1β and IL-12/23, but express high levels of immunosuppressive cytokines such as IL-10 and TGF-β, high arginase-1 activity and specific surface markers such as CD163 and CD206 (mannose receptor). Functional plasticity of macrophages during tumor progression has been proposed. Macrophages at early stages of tumor initiation show an M1 phenotype, while TAMs in established tumors show an M2-biased phenotype. As a particular pathophysiological consequence in the setting of cancer, the M1-M2 switch is the key step that accelerates tumor aggressiveness [2, 6, 7].

A large body of work describing a link between inflammation and cancer has generated intense interest in Cyclooxygenase-2 (COX-2) that is the rate-limiting enzyme in the metabolic conversion of arachidonic acid (AA) into various prostaglandins (PGs) including prostaglandin E_2_ (PGE_2_) for mediating inflammation and cancer progression. Over-expression of COX-2 has been detected in a number of malignancies including breast cancer, and contributes to carcinogenesis by stimulating cancer cell proliferation, inhibiting apoptosis, increasing invasiveness and modulating inflammation and immunity [8, 9]. Clinical studies have noted a reduced risk for breast, lung, prostate, and colon cancers after treatment with non-selectively COX-2 inhibition by non-steroidal anti-inflammatory drugs (NSAIDs) or selective COX-2 inhibition with COX-2 inhibitors [10, 11]. COX-2 and its products, particularly PGE_2_, act via classical cancer signaling pathways in primary tumor cells to promote tumorigenesis. Recent evidence has shed a spotlight not only on the tumor cell itself, but also the tumor microenvironment, especially macrophages in the tumor [12]. COX-2-positive TAMs are found in more advanced melanoma, and appear to act as a biomarker for melanoma progression [13]. TAMs in the post-irradiated tumor microenvironment express a higher level of COX-2, and promote early prostate cancer growth in mice [14]. Although these data suggest that COX-2 in TAMs might participate in cancer process, its exact role and mechanism of which has not been well elucidated. In this study, we aim to investigate the contribution of COX-2 in TAMs to breast cancer progression, and to explore the mechanisms underlying the process.

## RESULTS

### High COX-2 expression in TAMs in breast cancer

**Figure 1:**
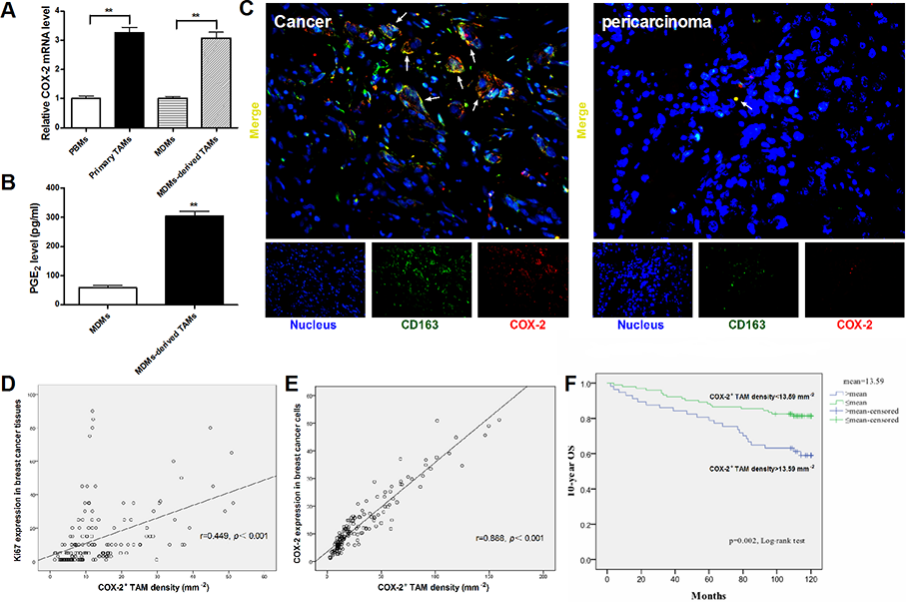
High COX-2 expression in breast cancer TAMs **A.** The relative COX-2 mRNA expression in different monocytes/macrophages. Mean ± SD, *n* = 9, **p < 0.01. **B.** PGE_2_ amount in supernatants of MDMs or MDMs-derived TAMs was measured by CIA assay. Mean ± SD, *n* = 9, **p < 0.01. **C.** The representative double immunofluorescence staining of CD163 (green) and COX-2 (red) in breast cancer tissues (Left) or pericarcinoma tissues (Right) (original magnification, × 400). **D.** Correlation of COX-2^+^ TAMs and Ki67 in breast cancer tissues (*n* = 160) was analyzed by Pearson's correlation analysis. **E.** Correlation of COX-2^+^ TAMs and COX-2 in breast cancer cells (*n* = 160) was analyzed by Pearson's correlation analysis. **F.** Kaplan-Meier 10-years OS curves for breast cancer patients according to COX-2^+^ TAMs density (*n* = 160).

Primary TAMs isolated from breast cancer tissue produced a large amount of IL-10 and arginase-1, and a small amount of IL-12/23, and exhibited a cluster of differentiation (CD)163^high^/CD206^high^ phenotype (Supplementary Figure S1). MDMs-derived TAMs established by *in vitro* co-culture of normal macrophages (monocyte-derived macrophages, MDMs) with breast caner cells for 7 days, also showed more M2-like characteristics than untreated MDMs (Supplementary Figure S1). Real-time PCR was performed to measure COX-2 expression in TAMs isolated from breast cancer patients, paired peripheral blood monocytes (PBMs), normal MDMs and MDMs-derived TAMs. The mRNA expression of COX-2 was significantly higher in primary TAMs and MDMs-derived TAMs, compared with that in PBMs and untreated MDMs (Figure 1A). Furthermore, MDMs-derived TAMs produced abundant amounts of PGE_2_ in the supernatants (Figure 1B). These results suggested that there was increased COX-2 expression and function in breast cancer TAMs.

### High COX-2 expression in TAMs correlates with poor prognosis in breast cancer patients

In order to determine the role of COX-2 in breast TAMs, a double immunofluorescent staining of COX-2 and CD163 (a specific marker for TAMs) was performed in a breast tissue array containing 160 human breast cancer tissue specimens and 10 pericarcinoma tissue controls. A greater number of COX-2^+^ macrophages were found in cancer samples than that in nonmalignant pericarcinoma samples (*p* < 0.001, Figure 1C). The number of COX-2^+^ TAMs was associated with increased clinical staging (*p* = 0.024) and aggressive tumor biology by advanced histopathological grading (*p* < 0.001) and lymph node metastasis (*p* = 0.021) (Table 1). Furthermore, there was a significant positive correlation between COX-2^+^ TAMs and the cell proliferation marker Ki67 (*r* = 0.449, *p* < 0.001, Figure 1D) or COX-2 expression (*r* = 0.888, *p* < 0.001, Figure 1E) in breast cancer cells. However, there was no association between COX-2^+^ TAM counts and other clinical parameters including patient age and molecular subtypes (*p* > 0.05). Kaplan-Meier survival curve with a median follow-up period of 118 months demonstrated that a significantly higher overall survival (OS) rate was observed in patients with low COX-2^+^ TAM counts than those with high COX-2^+^ TAM counts (*p* < 0.01, Figure 1F). In a multivariate Cox regression analysis, COX-2^+^ TAM counts were associated with poor survival prognosis of breast cancer patients (HR = 2.085, *p* = 0.036), independent of other clinical covariates (Table 2), indicating that COX-2^+^ TAM is an independent prognostic biomarker for breast cancer outcome, and COX-2 in TAMs may play an important role in breast cancer progression.

**Table 1:** Correlation of COX-2 Expressing TAM Counts with Clinicopathological Status in 160 Cases of Patients with Breast Cancer

Clinicopathological Status	COX2^+^TAMs (>13.59 mm^−2^, *n* = 57)	COX2^+^TAMs (≤ 13.59 mm^−2^, *n* = 103)	*p* Value
Age (years)	52 ± 14.68	54.50 ± 12.24	0.252
Tumor size (mm^3^)	60.16 ± 216.32	24.59 ± 44.34	0.225
TNM Stage			< 0.001
I	5(8.77%)	12(11.65%)	
II	20(35.09%)	76(73.79%)	
III	32(56.14%)	15(14.56%)	
Histological Grade			0.024
I	10(17.55%)	34(33.01%)	
II	42(73.68%)	68(66.02%)	
III	5(8.77%)	1(0.97%)	
Metastasis			0.021
Yes	46(80.70%)	47(45.63%)	
No	11(19.30%)	56(54.37%)	
ER			0.527
+	42(73.68%)	71(68.93%)	
−	15(26.32%)	32(31.07%)	
PR			0.479
+	37(64.91%)	61(59.22%)	
−	20(35.09%)	42(40.78%)	
HER2			0.067
+	14(24.56%)	40(38.83%)	
−	43(75.44%)	63(61.17%)	
Ki67			0.03
+(≤ 5%)	34(59.65%)	43(41.75%)	
−(>5%)	23(40.35%)	60(58.25%)	

**Table 2:** Multivariate Cox regression analysis of potential prognostic factors for breast cancer

clinical characteristics	Hazard ratio	95% CI	*P* values
Age, *y* > 60	1.694	0.902–3.181	0.101
Tumor size(>2 cm)	0.648	0.314–1.337	0.240
TNM stage (III)	2.015	1.032–3.933	0.040
Histological Grade (>II)	2.925	1.096–7.802	0.032
ER	0.798	0.418–1.532	0.494
PR	0.691	0.369–1.295	0.249
HER2	1.795	0.939–3.433	0.077
Ki67	0.902	0.488–1.668	0.743
Density of COX-2^+^ TAM (>13.59 mm^−2^)	2.085	1.050–4.140	0.036

### Over-expression of COX-2 in TAMs promotes breast cancer cell proliferation and survival

**Figure 2:**
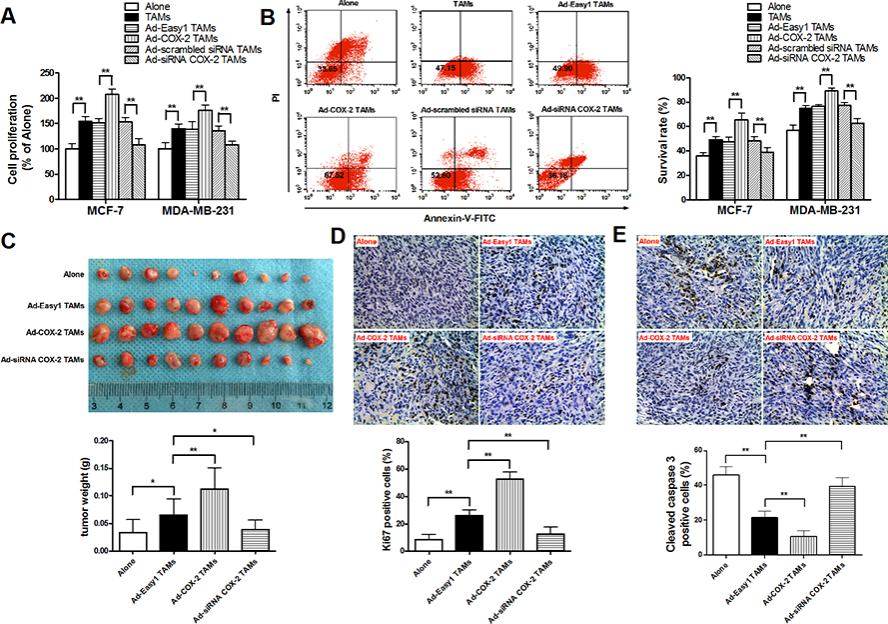
COX-2 in macrophages promotes breast cancer growth **A.** Cell proliferation assay. After breast cancer cells were co-cultured with or without TAMs transfected with adenoviral COX-2 or siRNA COX-2 for 7 days, cell proliferation was measured by CCK-8 kit. Data were expressed as a ratio of treated cells to control (Alone) cells. **B.** Cell apoptosis in breast cancer cells induced by adriamycin (ADM) was examined by flow cytometry analysis of Annexin V-FITC/PI staining. Numbers inside dot plots indicate the percentages of survival cells. **C.** 4T1 cells alone or with Raw264.7-derived TAMs transfected with adenoviral COX-2 or siRNA COX-2 were injected into the mammary fat pads of NOD/SCID mice. Tumor weight was measured in different groups. **D.** and **E.** Ki-67 staining for cell proliferation and cleaved caspase 3 for apoptosis were evaluated by IHC (original magnification, × 400). The number of stained and unstained cells was counted to generate the percentage of positive cells in each group. All the data were presented as the mean ± SD, *n* = 10, *p < 0.05 and **p < 0.01.

In order to elucidate the tumor-promoting role of COX-2 in breast TAMs, TAMs were first transfected with adenoviral COX-2 or siRNA COX-2 (Supplementary Figure S2), and then co-cultured with different breast cancer cell lines (MCF-7 and MDA-MB-231) for 7 days. Cancer cell proliferation, viability or apoptosis induced by various cytotoxic drugs were measured by CCK-8 or PI staining assays, respectively. We found that TAMs promoted proliferation and resistance to drugs-induced apoptosis in breast cancer cells, which was enhanced by COX-2 over-expression but attenuated by COX-2 knockdown in TAMs (Figure 2A–2B and Supplementary Figure S3). Consistent with these *in vitro* findings, higher mammary tumor weight/volume was observed in NOD/SCID mice injected with 4T1 murine breast cancer cells/RAW 264.7-derived TAMs, compared with that in mice injected with 4T1 cells only. Tumor weight/volume was much higher in mice injected with 4T1/COX-2^+^ TAMs, while lower in mice injected with 4T1/COX-2^−^ TAMs than that in mice injected with 4T1/normal TAMs (Figure 2C). Furthermore, significantly increased proliferation (Ki-67 staining) and decreased apoptosis (cleaved caspase 3 staining) were detected in the tumor specimens of mice injected with 4T1/COX-2^+^ TAMs, while an inverse result was obtained from mice injected with 4T1/COX-2^−^ TAMs, compared with that of mice injected with 4T1/normal TAMs (Figure 2D–2E).

### PGE_2_ is unlikely the only mediator of the effect of TAMs COX-2 on breast cancer cells

**Figure 3:**
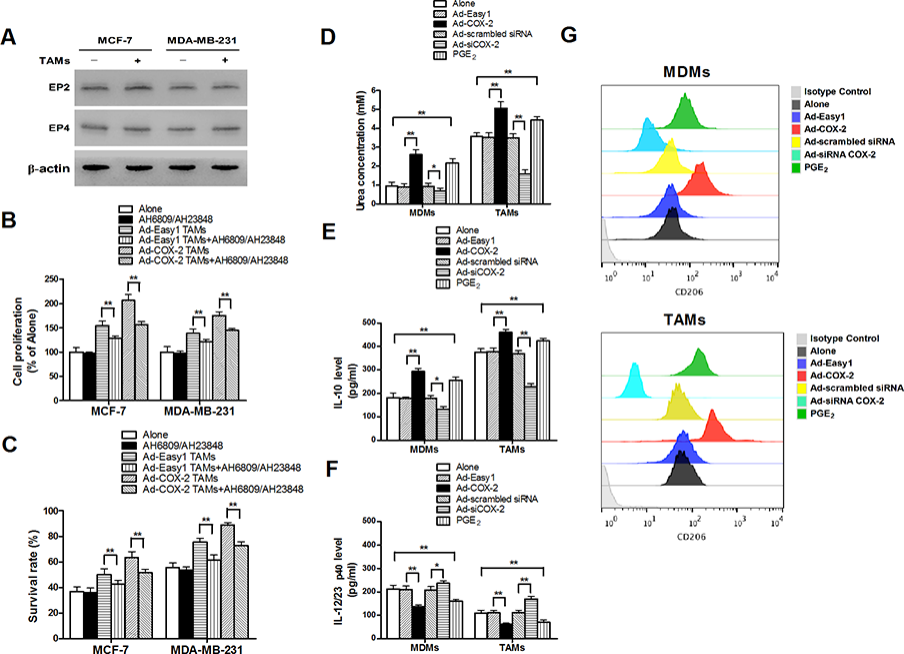
COX-2 is essential for macrophages polarized to M2 phenotype **A.** The expression of EP2 and EP4 in breast cancer cells co-cultured with or without TAMs was detected by Western blot. β-actin was used as an internal loading control. **B.** and **C.** Inhibiting PGE_2_ signal pathway partly attenuated cell proliferation and drug resistance induced by COX-2 in TAMs. Breast cancer cells were first treated with or without EPs antagonists AH6809 (5 μM) and AH23848 (10 μM) for 12 h before co-cultured with or without TAMs transfected with adenoviral COX-2. Cell proliferation (B) and cell apoptosis (C) induced by ADM were measured by CCK-8 kit and flow cytometry respectively. **D.** Arginase activity (urea concentration) in macrophages transfected with adenoviral COX-2 or siRNA COX-2 or treated with PGE_2_ (1 μM) was analyzed by microplate reader. **E.** and **F.** Expression of IL-10 and IL-12/23 in macrophages was detected by ELISA. **G.** Expression of CD206 in macrophages was analyzed by flow cytometry. All the experiments were performed thrice in triplicate. Mean ± SD, *p < 0.05 and **p < 0.01.

As the key factor for the biological function of the COX-2 pathway, PGE_2_ activates intracellular signal transduction by binding to the E-series of prostaglandin receptors EP1, EP2, EP3 and EP4. Increasing studies indicate that EP2 and EP4 are the main EP subtypes involved in mammary tumor progression [15]. In order to investigate whether COX-2 in macrophages exerted pro-tumor activity mainly through direct PGE_2_ effect on cancer cells, the expression of EP2 and EP4 in breast cancer cells was confirmed (Figure 3A). The antagonist against EP2 (AH6809) and EP4 (AH23848) were used to block the PGE_2_ signaling. The results showed that inhibiting the PGE_2_ signal pathway only partly attenuated cell proliferation and drug resistance in breast cancer cells induced by TAMs (Figure 3B–3C), suggesting that other mediators are involved in COX-2-mediated communication between TAMs and breast cancer cells.

### COX-2 is essential for macrophages polarized to M2 phenotype

Since the polarization of infiltrating macrophages to M2-like TAMs is the key step that promotes tumor development by inducing various tumor-related cytokines, we next investigated whether COX-2 in TAMs promoted cancer cell survival and proliferation by enhancing macrophages polarity. The expression of EP1–4 in MDMs and TAMs was confirmed (Supplementary Figure S4). The phenotypes of macrophages transfected with adenoviral COX-2 or siRNA COX-2 were then identified by detection of arginase activity and cytokines. We found that COX-2 over-expression or exogenous PGE_2_ promoted MDMs polarized to M2 phenotype. In contrast, TAMs treated with siRNA COX-2 lost M2 phenotype (Figure 3D–3G). These results sytrongly suggest that COX-2 is an essential factor for the induction and maintenance of M2 polarity in TAMs.

### COX-2 in TAMs increases the expression of Bcl-2 and P-glycoprotein and decreases Bax expression in breast cancer cells

**Figure 4:**
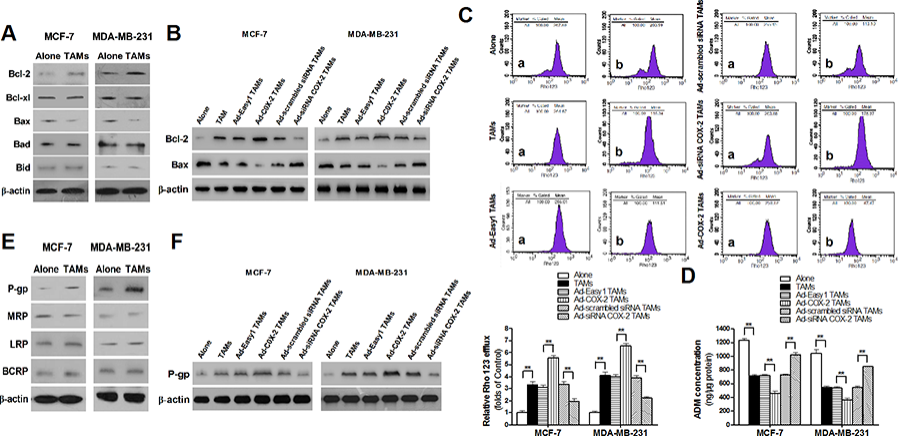
COX-2 in TAMs increases the expression of Bcl-2 and P-gp and decreases Bax expression in breast cancer cells **A.** The expression of Bcl-2 family members in breast cancer cells co-cultured with or without TAMs was detected by Western blot. **B.** The expression of Bcl-2 and Bax in breast cancer cells co-cultured with or without TAMs transfected with adenoviral COX-2 or siRNA COX-2 was detected by Western blot. **C.** Rho 123 efflux in breast cancer cells was analyzed by flow cytometry. The panel shows Rho 123 efflux in MDA-MB-231 cells. a, intake amount; b, residue amount. Data were expressed as a ratio of treated cells to control (Alone) cells. Mean ± SD, *n* = 9, **p < 0.01. **D.** The concentration of ADM in human breast cancer cells was measured by spectrophotometer. Mean ± SD, *n* = 9, **p < 0.01. **E.** The expression of MDR related proteins in breast cancer cells co-cultured with or without TAMs was detected by Western blot. **F.** The expression of P-gp in breast cancer cells co-cultured with or without TAMs transfected with adenoviral COX-2 or siRNA COX-2 was detected by Western blot. In all Western blot assays, β-actin was used as an internal loading control, and the blots shown are representative of six independent experiments.

Apoptosis is a well-orchestrated process regulated by multiple pro-apoptotic and anti-apoptotic factors, particularly the Bcl-2 family members. These factors are well documented in breast cancer, and aberrant expressions of which are strongly associated with cell survival or resistance to chemotherapeutic drugs [16, 17]. In order to identify whether or which Bcl-2 family members are involved in COX-2^+^ TAMs-induced breast cancer cell survival, the common anti-apoptotic proteins (Bcl-2 and Bcl-xl) and pro-apoptotic proteins (Bax, Bad and Bid) were detected by Western blot. TAMs induced Bcl-2 and decreased Bax expression in breast cancer cells (Figure 4A). Ectopic COX-2 expression significantly promoted, while COX-2 knockout in TAMs markedly abated, this effect (Figure 4B). In addition, TAMs increased the efflux of ADM and Rho 123 in cancer cells. Ectopic COX-2 expression in TAMs significantly reinforced, while inhibiting COX-2 in TAMs attenuated, this effect (Figure 4C–4D). Because the ATP-binding cassette (ABC) transporters including P-glycoprotein (P-gp/ABCB1), multi-drug resistance-associated protein 1 (MRP1/ABCC1), lung resistance protein (LRP), and breast cancer resistance protein (BCRP/ABCG2), are membrane proteins that couple the energy derived from ATP hydrolysis to extrude a variety of chemotherapeutic drugs out of the cancer cells [18], we examined the effect of COX-2 on expression of these proteins by Western blot. We found that P-gp but not other proteins was significantly increased in cancer cells co-cultured with TAMs (Figure 4E). The association of P-gp expression in breast cancer cells and COX-2 in TAMs was validated by ectopic COX-2 expression and COX-2 knockdown in TAMs (Figure 4F). These results suggest that TAMs COX-2 enhances breast cancer cell survival by increasing Bcl-2 and P-gp, and decreasing Bax in cancer cells.

### COX-2 in TAMs induces the expression of COX-2 in breast cancer cells, which in turn, promotes macrophage polarization to M2 phenotype

**Figure 5:**
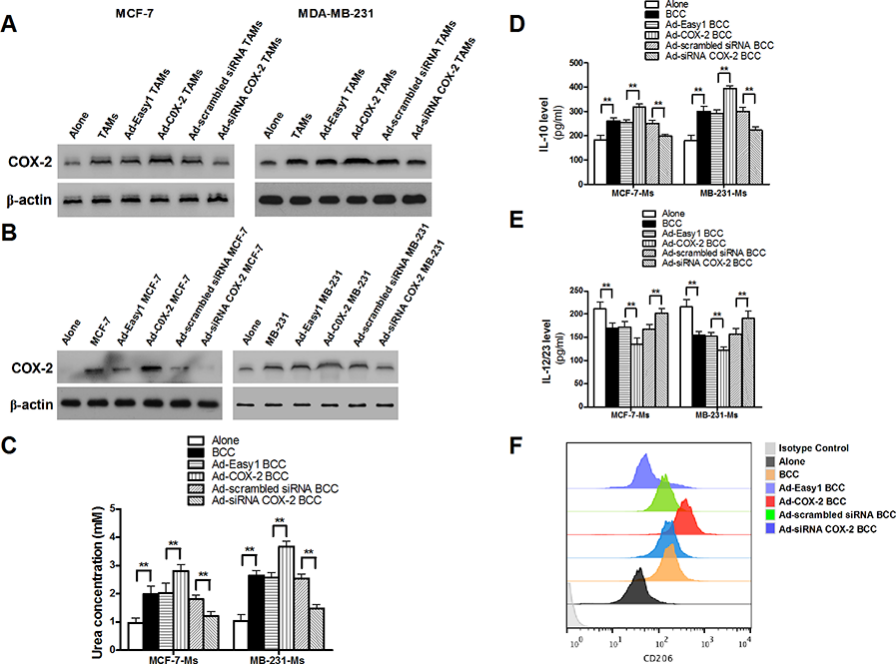
COX-2 in TAMs induces the expression of COX-2 in breast cancer cells, which in turn, promotes macrophage polarization to M2 phenotype **A.** The expression of COX-2 in breast cancer cells co-cultured with or without TAMs transfected with adenoviral COX-2 or siRNA COX-2 was detected by Western blot. **B.** The expression of COX-2 in macrophages co-cultured with or without breast cancer cells transfected with adenoviral COX-2 or siRNA COX-2 was detected by Western blot. In all Western blot assays, β-actin was used as an internal loading control, and the blots shown are representative of six independent experiments. **C.** Arginase activity (urea concentration) in macrophages co-cultured with or without breast cancer cells transfected with adenoviral COX-2 or siRNA COX-2 was analyzed by microplate reader. **D.** and **E.** Expression of IL-10 and IL-12/23 in macrophages was detected by ELISA. **F.** Expression of CD206 in macrophages co-cultured with breast cancer cells (MDA-MB-231 cells) was analyzed by flow cytometry. All the experiments were performed thrice in triplicate. Mean ± SD, *p < 0.05 and **p < 0.01.

Over-expression of COX-2 in tumor cells is closely linked to cell survival, and it is suggested that TAMs are associated with high COX-2 expression in cancer cells [19–21]. In addition, PGE_2_ is also demonstrated to activate COX-2 expression with a positive feedback manner [22, 23]. To investigate the mutual activation between COX-2 in different cell types, the expression of COX-2 in cancer cells and macrophages under different co-culture conditions was examined. We found that COX-2 over-expression in TAMs enhanced COX-2 expression in breast cancer cells, while inhibiting COX-2 in TAMs attenuated this COX-2 induction in cancer cells (Figure 5A). Interestingly, COX-2 in breast cancer cells was shown to activate COX-2 in macrophages and induce M2 macrophage polarization (Figure 5B–5F). These results suggest that COX-2 expressed in both TAMs and cancer cells forms a positive feedback loop for mediating communication between TAMs and cancer cells.

### COX-2 in TAMs enhances the activation of PI3K/Akt pathway in breast cancer cells

**Figure 6:**
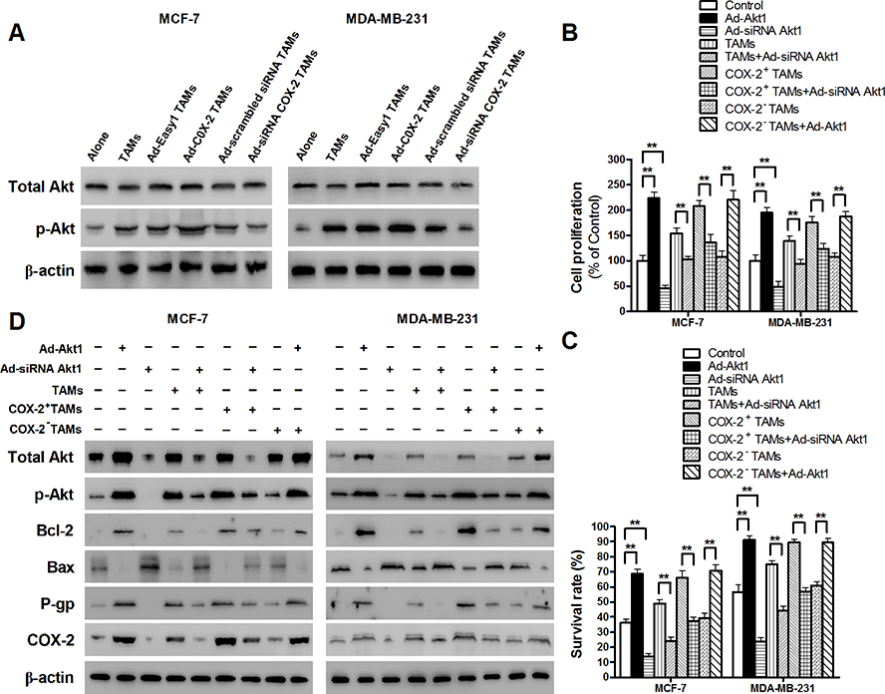
COX-2 in TAMs enhanced the activation of PI3K/Akt pathway in breast cancer cells **A.** The activation of Akt in breast cancer cells co-cultured with or without TAMs transfected with adenoviral COX-2 or siRNA COX-2 was detected by Western blot. **B.** and **C.** Inhibiting Akt1 in breast cancer cells attenuated cell proliferation and drug resistance induced by COX-2 in TAMs. Breast cancer cells transfected with adenoviral siRNA Akt1 or Akt1 were co-cultured with or without TAMs transfected with adenoviral COX-2 or siRNA COX-2. Cell proliferation (B) and cell apoptosis (C) induced by ADM were measured by CCK-8 kit and flow cytometry, respectively. **D.** Inhibiting Akt1 resulted in the suppression of Bcl-2, P-gp and COX-2, and increase of Bax expression in breast cancer cells co-cultured with COX-2^+^ TAMs. The expression of related proteins in breast cancer cells was detected by Western blot. β-actin was used as an internal loading control, and the blots shown are representative of six independent experiments.

TAMs activate PI3K/Akt pathway in cancer cells, and aberrant activation of the PI3K/Akt pathway is implicated in breast cancer cell proliferation and drug resistance [24–27]. Our results confirmed the activation of PI3K/Akt pathway in breast cancer cells co-cultured with TAMs. Moreover, COX-2 over-expression in TAMs enhanced the activation of PI3K/Akt pathway in breast cancer cells, while inhibiting COX-2 by siRNA suppressed this effect (Figure 6A). Among, the three Akt isoforms, Akt1 is the most widely expressed and the best studied one in cancer progression [28–30]. Blocking Akt1 in cancer cells suppressed the pro-survival effects of the COX-2^+^ TAMs on breast cancer cells (Figure 6B–6C). Meanwhile, down-regulation of Akt1 resulted in the suppression of Bcl-2, P-gp and COX-2, and increase of Bax expression in breast cancer cells (Figure 6D). These results strongly suggest that activation of the PI3K/Akt pathway is closely involved in COX-2^+^ TAMs-induced pro-tumor activity in breast cancer cells.

## DISCUSSION

TAMs have been shown to promote tumor growth, angiogenesis, invasion and metastasis in many cancer types. TAMs are generally thought to more closely resemble the M2-polarized phenotype [2, 6]. Here, we demonstrate that COX-2 is abundantly expressed in breast TAMs and correlates with poor prognosis in patients with breast cancer. COX is an enzyme that converts arachidonic acid into prostaglandin endoperoxide. Two isoforms of COX have been described. COX-1 is constitutively expressed in normal adult human tissues. In contrast, COX-2 is absent or expressed at low levels in normal human tissues, but is induced in endothelial cells, monocytes, and tumor cells by cytokines, growth factors, hormones, or tumor promoters [31]. Accumulating evidence shows that COX-2 is over-expressed in breast cancer cells and is closely associated with cell proliferation, metastasis, angiogenesis and immune regulation [32, 33]. In addition, high level of COX-2 was also prevalent in other components of tumor microenvironment such as fibroblasts, myeloid cells and vascular endothelial cells, and may contribute to malignant tumor progression [34–36]. In our study, we found that over-expression of COX-2 in breast TAMs promoted breast cancer cell proliferation and survival. More importantly, COX-2 exerted an effect on the pro-tumor potential of TAMs. These results indicate that high expression of COX-2 in the tumor microenvironment exerts great influence on cancer progression.

As the principle COX-2 product, PGE_2_ plays a critical role in the biological function of COX-2 in tumors. PGE_2_ has been implicated in various breast cancer processes via activating PKC, PKA or TGF-β signal pathways through specific PGE_2_ receptors [15]. However, our study showed that inhibiting the PGE_2_ signal transduction in breast cancer cells only partly attenuated pro-tumor effect induced by COX-2 in TAMs, suggesting that PGE_2_ was not the only direct mediator involved in the process. It was reported that COX-2 inhibition caused loss of the M2 macrophage characteristics in TAMs, which may assist to prevent breast cancer metastasis in a murine breast cancer model [37]. Furthermore, PGE_2_ is typically associated with immunosuppression, restraint of M1 macrophage polarization, as well as enhanced expression of M2 markers [38–40]. Consistent with the reports, we confirmed that COX-2 was indeed an essential factor for the induction and maintenance of M2 polarity in human breast TAMs. COX-2 in TAMs may also exert its pro-tumor effect by increasing various pro-tumor cytokines release from TAMs. Further studies are needed to identify the involved cytokines and elucidate the underlying mechanisms.

TAMs have been shown to induce cell proliferation and inhibit apoptosis in many kinds of malignant tumors such as sarcoma, lymphoma, colon and breast cancers [4, 41–43]. Enhanced stemness of cancer cells by cytokines (e.g. TGF-β and IL-1β) from TAMs may contribute to the pro-tumor activities of TAMs [43, 44]. In addition, TAMs can promote cell survival and resistance to chemotherapeutic treatment through regulating Bcl-2 family members and drug-resistance factors in cancer cells [45, 46]. In our study, we found that TAMs induced breast cancer cell survival by up-regulating Bcl-2 and p-gp and down-regulating Bax expression. It has been shown that COX-2 can regulate the expression of Bcl-2 family members and drug-resistance proteins in breast cancer cells [47–50]. Our study indicated that COX-2 in TAMs was involved in the regulating function of TAMs on Bcl-2 family members and p-gp in cancer cells. Moreover, we found that there was a positive COX-2-mediated feedback loop between TAMs and breast cancer cells, which may play a key role in tumor microenvironment reprogramming and thus cancer progression.

PI3K/Akt signaling pathway promotes cancer cell survival by modulating Bcl-2 members and drug-resistance proteins. Bcl-2, Bax and p-gp are all regarded as the downstream signal molecules of the PI3K/Akt pathway [51, 52]. The activation of PI3K/Akt pathway in cancer cells is a central event in TAMs-mediated cancer progression, as chemokines or cytokines secreted from TAMs may be the effective PI3K/Akt activators [24, 26, 53]. PGE_2_ was reported to inhibit apoptosis in human cancer cells through PI3K/Akt activation. Furthermore, COX-2/PGE_2_ was shown to promote the activation of PI3K/Akt pathway that is a part of the positive feedback loop to maintain an active pro-survival COX-2/PGE_2_ pathway in cancer cells [54–57]. Additionally, other cytokines (TGF-β, IL-10, CCL18, etc.) which can be released from TAMs also have the ability to promote tumor progression through the PI3K/Akt pathway [58–60]. All these factors could contribute to COX-2^+^ TAMs-induced PI3K/Akt activation in breast cancer cells, which warrants further studies.

**Figure 7:**
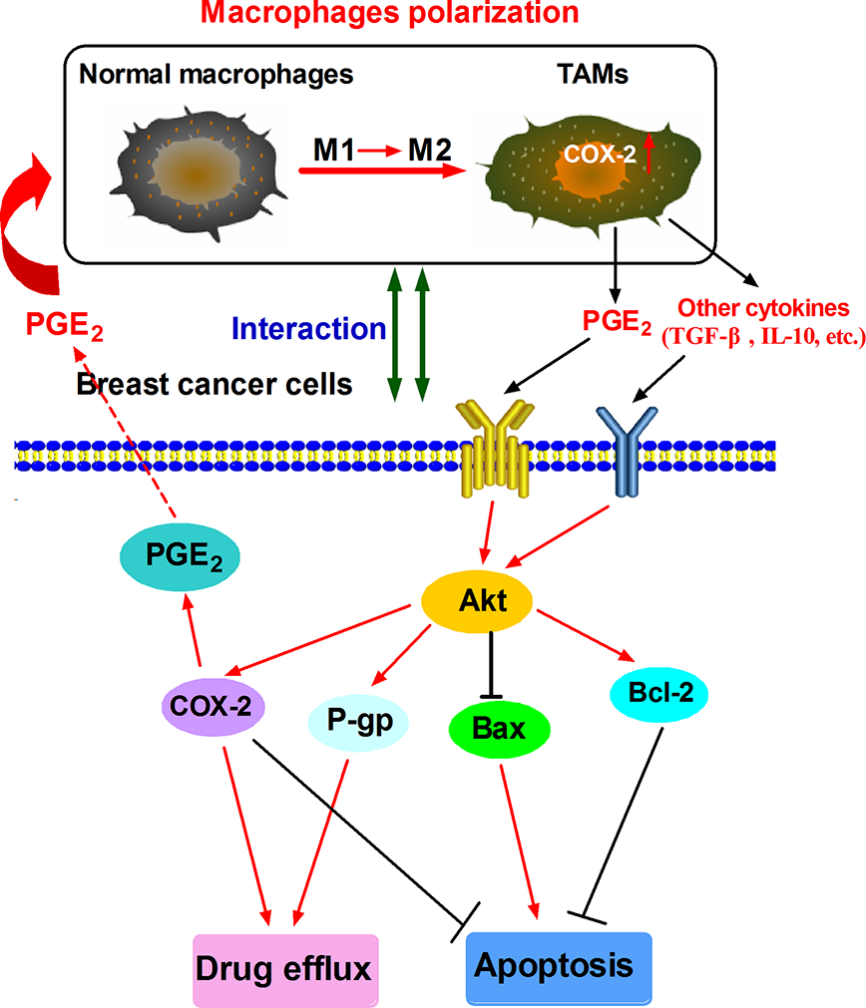
Proposed mechanistic model showing COX-2^+^ TAMs-induced cell survival in breast cancer

In summary, our studies shows that COX-2, abundantly expressed in TAMs, is important for macrophages polarization and breast cancer cell survival (Fig. 7). Future studies are needed to investigate the detailed mechanism by which COX-2/PGE_2_ influences the tumor microenvironment. Accumulating evidence suggests COX-2 inhibitors are potential anticancer therapeutic agents [61]. Studies on the emerging roles within the COX-2/PGE_2_ pathway in tumor microenvironment may reveal novel approaches or molecular targets for both cancer chemoprevention and therapy.

## MATERIALS AND METHODS

### Clinical data collection

Breast cancer tissue microarrays (HBre-Duc 170Sur-01, Outdo Biotech Co., Shanghai, China) include primary breast carcinoma samples from 160 patients with a median age of 53 years and a median follow-up period of 118 months at the hospitals in central China. Every sample dot with a diameter of 1.5 mm and a thickness of 4 μm was prepared according to a standard method. All samples were collected with informed consent from patients, and all related procedures were performed with the approval of ethics boards of the indicated hospitals.

### Generation of macrophages or tumor associated macrophages

Mononuclear cells from the blood of healthy donors were incubated in 6-well plates for 2 hours at 37°C to remove non-adherent cells. The adherent monocytes were incubated for 7 days in medium with M-CSF to become normal macrophages (monocyte-derived macrophages, MDMs). MDMs were co-cultured with breast cancer cells for an additional 7 days to generate tumor associated macrophages (TAMs) [46].

### Isolation of TAMs from tumor tissues by magnetic-activated cell sorting

Tumor tissues were minced into small pieces and then incubated for 1 h at 37°C in 5 ml of HBSS (10% fetal calf serum) containing 125 U/ml of collagenase I (Invitrogen, CA, USA), 60 U/ml of Dnase I and 60 U/ml of hyaluronidase (Sigma, St Louis, MO, USA). Supernatants were harvested and then depleted of RBC with ACK. Cell suspensions were passed through a fine screen mesh and then several times through a ^#^25 needle. Cell pellets were resuspended and labeled with biotinylated mouse anti-human CD163 antibody (Abcam, Cambridge, MA, USA) using 0.2 μg/1 × 10^6^ cells in 100 ml staining buffer. Streptavidin microbeads were added at 10 ml per 10^7^ cells and then incubated 15 min at 4°C. Cells were re-suspended in 500 ml of separating buffer and applied to an LS column in a magnetic field (Miltenyi Biotec, Bergisch Gladbach, Germany). Unlabeled cells that passed through the column were collected, as were retained cells after removal of the magnetic field [62].

### Arginase activity assay

The arginase activity was measured as previously described [63]. Briefly, the cell lysate was activated by heating for 10 min at 56°C. L-arginine hydrolysis was conducted by incubating the activated lysate with 0.5 M L-arginine (pH 9.7) at 37°C for 15 to 120 min. The reaction was stopped with H_2_SO4(96%)/H_3_PO4(85%) /H_2_O (1:3:7, v/v/v, VWR). α-isonitrosopropiophenone (ISPF) dissolved in 100% ethanol (Sigma, St Louis, MO, USA) was added and incubated for 45 min at 100°C, followed by 30 min at 4°C. A standard curve was obtained by treating serially diluted urea with ISPF and incubated in the final step. The optical density (OD) was measured at 550 nm. Protein concentration of samples was measured using the BCA Protein Assay kit (Pierce, Rockford, USA). One unit of enzyme activity is defined as the amount of enzyme that catalyzes the formation of 1 μmol of urea per min.

### ELISA and EIA

The levels of IL-10 and IL-12/23 p40 in macrophages were determined by ELISA using human ELISA Kits, according to the manufacturers’ instruction (R&D Systems, Minneapolis, MN, USA). The PGE_2_ amount in macrophages was determined by EIA kits, according to the manufacturers’ instruction (Cayman Chemical, Ann Arbor, Michigan USA).

### Quantitative reverse transcription polymerase chain reaction

Real-time PCR was carried out in ABI 7500 Real-Time PCR System (Applied Biosystems) by using Maxima SYBR Green/ROX qPCR Master Mix (MBI Fermentas, St. Leon-Rot, Germany). Primer pairs were as follows: (a) COX-2, 5′-TCCCTGAGCATCTACGGTT-3′ (forward) and 5′-CATCGCATACTGTTGTGTTC -3′ (reverse); and (b) β-actin, 5′-CCTGTGGCATCCACGAAACT-3′ (forward) and 5′-GAAGCATTTGCGGTGGACGAT-3′ (reverse), encoding products of 185 and 314 bp, respectively. Thermal cycling conditions were 95°C for 30 sec, followed by 5 sec at 95°C, 1 min at 60°C for 40 cycles. Melting-curve analysis and agarose gel electrophoresis of PCR products were further performed. Relative expression levels of COX-2 in cells were standardized to β-actin levels.

### Adenovirus infection

The adenovirus expressing empty Ad-Easy1 vector, COX-2, Akt1, scrambled siRNA or siRNA COX-2 or Akt1 was used following the procedure described previously [64]. Target sequences of the siRNA specific for COX-2 and Akt1 are as follows: siRNA-COX-2, 5′-AACCGAGGTGTATGTATGAGTGT-3′; siRNA-Akt1, 5′-TCGTGCCATGATCTGTATTTAAT-3′. In addition to the expression of transgenes, the adenovirus expressing system also expressed RFP as amarker for monitoring transfection efficiency. A series of infections using various dilutions of adenovirus were conducted to determine the optimal multiplicity of infection (MOI) in which expression of target genes occurred with low cytotoxicity.

### Cell proliferation/viability assay

Cells seeded into a 96-well plate at 4000 cells/well were treated with or without different concentrations of chemotherapeutic drugs and incubated for 48 h. Cell viability was measured according to the protocol of CCK-8 (KeyGEN Biotech, Nanjing, China). All plates had control wells containing medium without cells to obtain *a* value for background spectrometric absorbance which was subtracted from the test sample readings. Data were expressed as ratios of treated to control cells, mean ± SD for three replications.

### Flow cytometry analysis of cell apoptosis

For apoptosis analysis, Annexin V-FITC/propidium iodide (PI) staining (KeyGEN Biotech, Nanjing, China) was performed by Elite ESP flow cytometry according to the manufacturer's guidelines.

### Animal experiments

All the animal studies were approved by the Animal Ethics Committee of Chongqing Medical University. 5-Week old severe combined immunodeficiency (SCID) hairless female mice were purchased (Institute of Laboratory Animal Science, Chinese Academy of Medical Science, Beijing, China) and randomly divided into four groups of 10 mice each. All the mice were housed according to the national and institutional guidelines for humane animal care. Macrophages in mice were depleted by injection of freshly prepared clodronate-containing liposomes as described [62]. At 6 weeks of age, the mice were injected subcutaneously on the right rear flanks with 4T1 cells that were prior admixed with COX-2 over-expression, COX-2 knockdown or wild-type RAW264.7-derived TAMs, respectively. Body weights were monitored weekly as an indicator of overall health. After 4 weeks, the mice were euthanized via CO_2_ asphyxiation. Tumors were then removed, weighed, and sent for immunohistochemistry (IHC) analysis.

### Indirect immunofluorescence analysis

For the immunofluorescence experiments, paraffin embedded tissues were prepared and analyzed under fluorescence microscope following the procedure described previously [65]. Briefly, samples were incubated with primary mouse antibody against CD163 and rabbilt antibody against COX-2, and then incubated with DyLight 488 against mouse IgG or DyLight 549 secondary antibody against rabbit IgG (Cwbiotech, Beijing, China). Cells were then counterstained with DAPI and imaged with a fluorescence microscope (Leica DM IRB).

### IHC

Tumor tissues were fixed in 4% formaldehyde solution (pH 7.0) and subsequently embedded in paraffin. Immunohistochemical studies were performed using the standard streptavidin-peroxidase (SP) method with the UltraSensitive TM SP Kit (Maixin-Bio, Fujian, China) according to the manufacturer's instructions. Tumor specimens were stained using Ki-67 antibody (Maixin-Bio, Fujian, China) for cell proliferation and cleaved caspase 3 antibody (Cell Signaling Technology, Inc., Danvers, MA) for apoptosis. Negative control was performed by replacing the primary antibody with PBS. Immunostained slides were blindly evaluated by a trained pathologist under a transmission light microscope.

### Western blot

Cell lysate was prepared according to the method described by the protein extract kit (Active Motif Company, Carlsbad, USA). Protein concentrations were determined by BCA protein assay kit (Pierce Biotechnology Inc, Rockford, USA). Cell lysate was analyzed for Western blot analysis using EP1, EP2, EP3, EP4, Bcl-2, Bcl-xl, Bax, Bad, Bid, P-gp, MRP1, LRP, BCRP, COX-2, total and phosphor(p)-Akt plus β-actin (Detailed information about antibodies is shown in Supplementary Table S1). Antibody binding was visualized with an ECL chemiluminescence system and short exposure of the membrane to X-ray films (Kodak, Japan). Densitometric analysis was done using Image Pro-Plus software and normalized to β-actin.

### Rhodamine (Rho) 123 efflux assay

Rho123 was added to 1 × 10^6^/ml cells at the final concentration of 1 μg/ml, and incubated at 37°C for 1 h. After washing with PBS for 3 times, intracellular Rho123 was determined by flow cytometry. The cells above washed by PBS for 3 times were resuspended and then cultured with 1640 medium without Rho123 for 2 h. After washing with PBS for 3 times, intracellular Rho123 was determined by flow cytometry.

### Intracellular adriamycin accumulation assay

Cells were first incubated with 1640 medium containing 10 μg/ml ADM at 37°C for 1 h. After washing with PBS for 3 times, the cells were resuspended and then cultured with 1640 medium without adriamycin (ADM) for 2 h. After washing with PBS for 3 times, intracellular ADM was measured by fluorescence spectrophotometer.

### Statistical analysis

All statistical analysis was done using SPSS 18.0 software. Chi-square test was applied to analyze the relationship between COX-2^+^ TAMs counts and clinicopathological status. Pearson's correlation and regression analysis was performed to assess the relationship between COX-2^+^ TAMs and Ki-67 or COX-2 in the enrolled samples. Kaplan-Meier survival curves were plotted, and log rank test was done. The significance of various variables for survival was analyzed by the Cox proportional hazards model in a multivariate analysis. The data in cell experiments were presented as the mean values ± standard deviation (SD). Differences were considered significant when the *p* values were 0.05.

## SUPPLEMENTARY FIGURES AND TABLE


