# Supplementary material for: Cyclooxygenase-2 in tumor-associated macrophages promotes breast cancer cell survival by triggering a positive-feedback loop between macrophages and cancer cells
Source: Oncotarget. 2015 Aug 10;6(30):29637–50. doi: 10.18632/oncotarget.4936 (PMC4745752; doi:10.18632/oncotarget.4936)
Supplement: Supplementary file 1 [file oncotarget-06-29637-s001.pdf]

SUPPLEMENTARY FIGURES AND TABLE

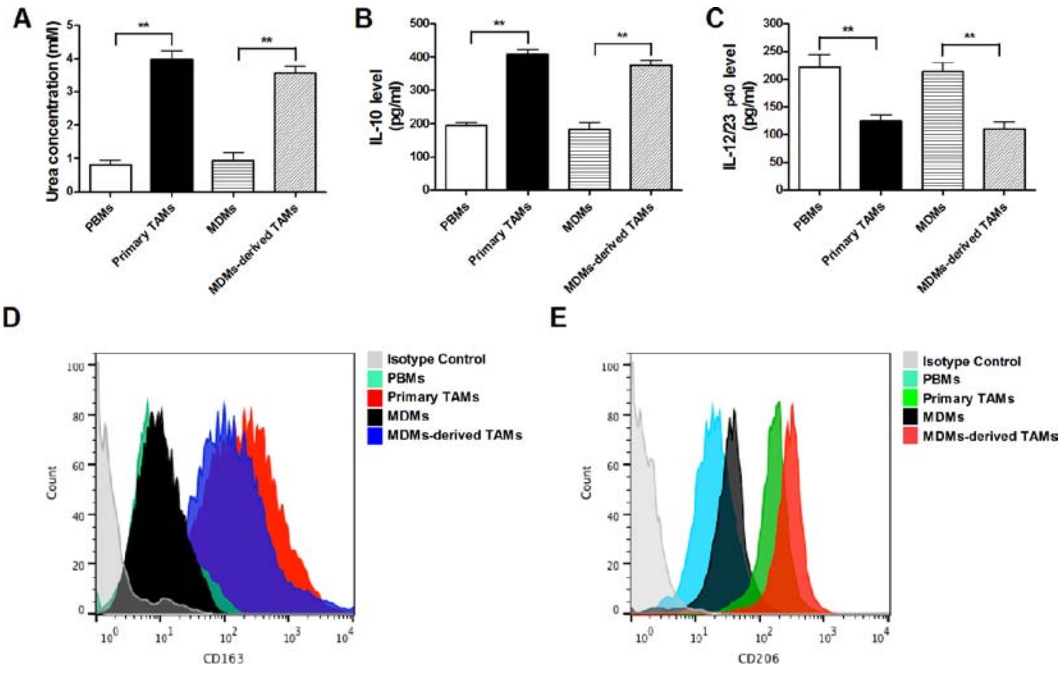

Supplementary Figure S1: The molecular phenotype of macrophages in human breast cancer or co-cultured with breast cancer cells. A. Arginase activity (urea concentration) in peripheral blood monocytes (PBMs), primary tumor-associated macrophages (TAMs), monocyte-derived macrophages (MDMs), or MDMs-derived TAMs. **B.** and **C.** Expression of IL-10 and IL-12/23 in macrophages was detected by ELISA. **D.** and **E.** Expression of CD163 and CD206 in macrophages was analyzed by flow cytometry. All the experiments were performed thrice in triplicate. Mean ± SD, *$p < 0.05$ and **$p < 0.01$.

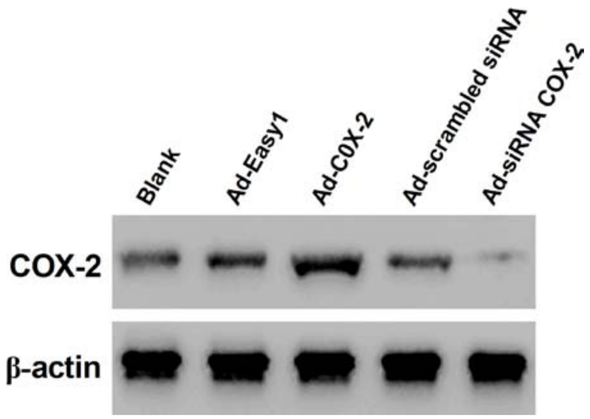

Supplementary Figure S2: Western blot analysis of COX-2 in TAMs. TAMs were transfected with control Ad-Easy1, Ad-COX-2, control Ad-scrambled siRNA or Ad-siRNA COX-2 for 48 h. COX-2 protein was detected by western blot. β-actin was used as a loading control, and the blots shown are representative of six independent experiments.

A **MCF-7**

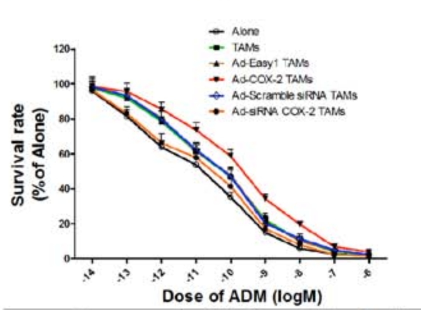

Groups		IC50 (ADM)	Relative resistance index
MCF-7	Alone	$(1.49\pm0.23)\times10^{-11}$	1
	TAMs	$(5.19\pm0.41)\times10^{-11}$	3.48
	Ad-Easy1 TAMs	$(5.69\pm0.36)\times10^{-11}$	3.81
	Ad-COX-2 TAMs	$(1.64\pm0.31)\times10^{-10}$	11.02
	Ad-scramble siRNA TAMs	$(5.63\pm0.28)\times10^{-11}$	3.77
	Ad-siRNA COX -2 TAMs	$(2.24\pm0.34)\times10^{-11}$	1.50

B **MDA-MB-231**

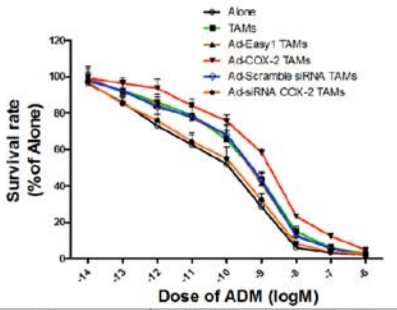

Groups		IC50 (ADM)	Relative resistance index
MDA-MB-231	Alone	$(4.55\pm0.38)\times10^{-11}$	1
	TAMs	$(2.05\pm0.45)\times10^{-10}$	4.51
	Ad-Easy1 TAMs	$(1.83\pm0.32)\times10^{-10}$	4.03
	Ad-COX-2 TAMs	$(6.54\pm0.39)\times10^{-10}$	14.37
	Ad-scramble siRNA TAMs	$(1.86\pm0.42)\times10^{-10}$	4.10
	Ad-siRNA COX -2 TAMs	$(5.99\pm0.36)\times10^{-11}$	1.32

C **MCF-7**

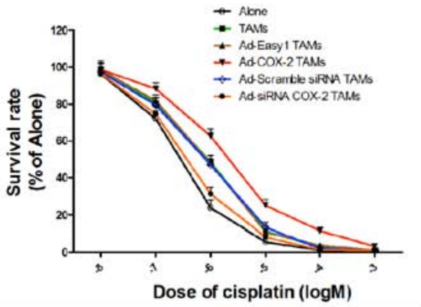

Groups		IC50 (Cisplatin)	Relative resistance index
MCF-7	Alone	$(4.69\pm0.32)\times10^{-7}$	1
	TAMs	$(1.16\pm0.29)\times10^{-6}$	2.46
	Ad-Easy1 TAMs	$(1.16\pm0.24)\times10^{-6}$	2.46
	Ad-COX-2 TAMs	$(2.62\pm0.37)\times10^{-6}$	5.57
	Ad-scramble siRNA TAMs	$(1.08\pm0.15)\times10^{-6}$	2.31
	Ad-siRNA COX -2 TAMs	$(6.23\pm0.39)\times10^{-7}$	1.33

D **MDA-MB-231**

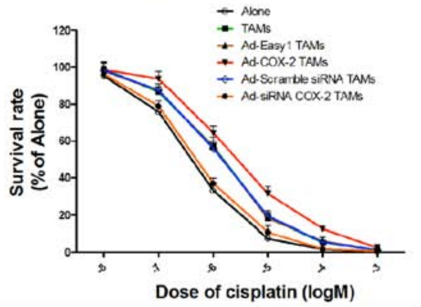

Groups		IC50 (Cisplatin)	Relative resistance index
MDA-MB-231	Alone	$(6.35\pm0.44)\times10^{-7}$	1
	TAMs	$(1.82\pm0.28)\times10^{-6}$	2.86
	Ad-Easy1 TAMs	$(1.78\pm0.30)\times10^{-6}$	2.80
	Ad-COX-2 TAMs	$(3.35\pm0.31)\times10^{-6}$	5.28
	Ad-scramble siRNA TAMs	$(1.8\pm0.22)\times10^{-6}$	2.84
	Ad-siRNA COX -2 TAMs	$(8.05\pm0.37)\times10^{-7}$	1.27

E **MCF-7**

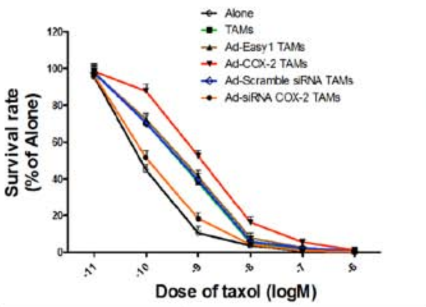

Groups		IC50 (Taxol)	Relative resistance index
MCF-7	Alone	$(1.36\pm0.23)\times10^{-10}$	1
	TAMs	$(6.54\pm0.49)\times10^{-10}$	4.82
	Ad-Easy1 TAMs	$(7.84\pm0.41)\times10^{-10}$	5.52
	Ad-COX-2 TAMs	$(1.62\pm0.25)\times10^{-9}$	11.94
	Ad-scramble siRNA TAMs	$(6.66\pm0.42)\times10^{-10}$	4.91
	Ad-siRNA COX -2 TAMs	$(2.21\pm0.38)\times10^{-10}$	1.63

F **MDA-MB-231**

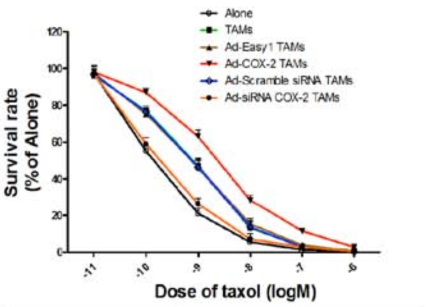

Groups		IC50 (Taxol)	Relative resistance index
MDA-MB-231	Alone	$(2.86\pm0.36)\times10^{-10}$	1
	TAMs	$(1.01\pm0.28)\times10^{-9}$	354
	Ad-Easy1 TAMs	$(1.04\pm0.20)\times10^{-9}$	363
	Ad-COX-2 TAMs	$(2.69\pm0.31)\times10^{-9}$	939
	Ad-scramble siRNA TAMs	$(9.92\pm0.42)\times10^{-10}$	346
	Ad-siRNA COX -2 TAMs	$(3.72\pm0.37)\times10^{-10}$	130

Supplementary Figure S3: COX-2 in macrophages enhances multi-drug resistance in breast cancer cells. Breast cancer cells which were co-cultured with or without TAMs transfected with adenoviral COX-2 or siRNA COX-2 for 7 days were treated with different concentrations of chemotherapeutic drugs including ADM (A and B), cisplatin (C and D) and taxol (E and F) and incubated for 48 h. At the end of the drug exposure duration, cell viability was measured according to the protocol of CCK-8 (KeyGEN Biotech, Nanjing, China). All plates had control wells containing medium without cells to obtain a value for background spectrometric absorbance which was subtracted from the test sample readings. Data were expressed as ratios of treated to control cells, mean ± SD for three replications.

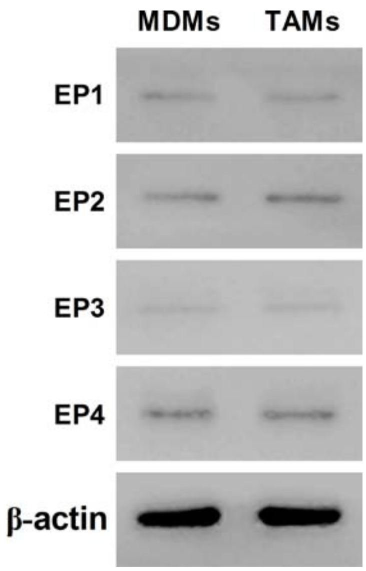

Supplementary Figure S4: Western blot analysis of EP1–4 in macrophages. EP1–4 proteins in MDMs and TAMs were detected by western blot. β-actin was used as a loading control, and the blots shown are representative of six independent experiments.

Supplementary Table S1: Antibodies used in this study

Antibody	Clone	Company	Isotype/Source	Dilution
Ki-67	MX006	Maixin-Bio	Mouse IgG	1:200
cleaved caspase 3	5A1E	Cell Signaling Technology	Rabbit IgG	1:200
Bcl-2	50E3	Cell Signaling Technology	Rabbit IgG	1:1000
Bcl-xl	54H6	Cell Signaling Technology	Rabbit IgG	1:1000
Bax	D2E11	Cell Signaling Technology	Rabbit IgG	1:1000
Bad	D24A9	Cell Signaling Technology	Rabbit IgG	1:1000
Bid	7A3	Cell Signaling Technology	Mouse IgG	1:1000
P-gp	EPR10364–57	Abcam	Rabbit IgG	1:2000
MRP1	EPR4658(2)	Abcam	Rabbit IgG	1:3000
LRP	EPR13227(B)	Abcam	Rabbit IgG	1:3000
BCRP	EPR2099(2)	Abcam	Rabbit IgG	1:2000
COX-2	D5H5	Cell Signaling Technology	Rabbit IgG	1:1000
total Akt	C67E7	Cell Signaling Technology	Rabbit IgG	1:1000
p-Akt (Ser473)	D9E	Cell Signaling Technology	Rabbit IgG	1:1000
EP1	Polyclonal	Abcam	Rabbit	1:1000
EP2	EPR8030(B)	Abcam	Rabbit IgG	1:2000
EP3	5F5	Abcam	Mouse IgG	1:1000
EP4	Polyclonal	Abcam	Rabbit	1:1000
β-actin	D6A8	Cell Signaling Technology	Rabbit IgG	1:1000
